# Lead Toxicoses in Free-Range Chickens in Artisanal Gold-Mining Communities, Zamfara, Nigeria

**DOI:** 10.5696/2156-9614-10.26.200606

**Published:** 2020-05-26

**Authors:** Olusola O. Oladipo, Olatunde B. Akanbi, Pius S. Ekong, Chidiebere Uchendu, Oyetunji Ajani

**Affiliations:** 1 Biochemistry Division, National Veterinary Research Institute, Vom, Nigeria; 2 Department of Veterinary Physiology, Biochemistry and Pharmacology, University of Jos, Jos, Nigeria; 3 Department of Veterinary Pathology, University of Ilorin, Ilorin, Nigeria; 4 Central Diagnostics Division, National Veterinary Research Institute, Vom, Nigeria; 5 Department of Veterinary and Pest Control Services, Federal Ministry of Agriculture & Rural Development, Abuja, Nigeria

**Keywords:** mining, gold mining, lead, toxicity, chickens, Zamfara, Nigeria

## Abstract

**Background.:**

In early 2010, outbreaks of lead poisoning due to artisanal gold mining in villages in the northwest Nigerian state of Zamfara have resulted in the death of hundreds of children < 5 years old. There have also been unconfirmed reports of high mortality of geese within these villages.

**Objectives.:**

To report a case of lead poisoning in three domestic free-range chickens found in one of the affected communities where illegal small-scale gold mining activities take place.

**Methods.:**

Three free-range domestic chickens were presented during a field investigation in one of the villages. The birds were observed to be emaciated, weak, showing nervous manifestations and moribund.

**Results.:**

Tissue extracts of liver, spleen and intestines were negative for Newcastle viral antigens, while cultures of liver and spleen biopsy were positive for Escherichia coli. Histopathological lesions were observed in the kidney, proventriculus and brain. Concentrations of lead in the tissues ranged between 7.5 mg/kg and 120.5 mg/kg wet weight, and the potential daily intake of lead in the tissues were estimated at 34.06–200.15 μg/day/kg body weight with an average of 118.37 μg/day/kg body weight.

**Conclusions.:**

The results of the present study suggest probable risk to human health due to the consumption of chicken contaminated by lead in the affected villages. Poisoning in animal populations may serve as a sentinel to assess the extent of environmental contamination and human health problems related to lead.

**Ethics Approval.:**

Protocols were approved and performed in accordance with relevant local guidelines and regulations as set by the Animal Care and Use Committee of the National Veterinary Research Institute, Vom, Nigeria.

**Competing Interests.:**

The authors declare no competing financial interests.

## Introduction

Mining activities are known to increase the bioavailability of metals in aquatic and terrestrial ecosystems and often leave a legacy of environmental pollution long after active mining has ceased.^1^,^2^ Lead (Pb), a well-known environmental toxicant, is a ubiquitous metal pollutant which is widespread in industrial areas. Humans and animals are exposed to Pb through food and feeds, as well as from the general environment.^3^ Lead toxicity has been shown to cause mortality in birds.^4^ Sources of Pb exposure in birds include Pb bullet shot, contaminated feed, water and soil, industrial pollution and agricultural technology.^5^ The adverse effects of Pb range from slight biochemical or physiological disorders to serious pathological conditions in which some organs and systems can be damaged or have their functions altered, according to the degree of exposure.^6^

In early 2010, environmental Pb contamination as a result of artisanal mining was reported in some northwest Nigerian villages.^7^–^9^ Mine run-off is also associated with contamination of the environment with Pb and other metals.^10^ Birds exposed to high concentrations of Pb will develop clinical symptoms quickly, with the majority of deaths linked to direct consumption of spent Pb shot through bullet fragments embedded in food items.^6^

This paper reports a case of lead poisoning in three domestic free-range chickens found in one of the affected communities where illegal small-scale gold mining activities take place. This community, and other similar communities have recorded high animal and human mortality due to lead, especially in children <5 years of age.^9^,^11^,^12^ This report details part of the findings from a larger study conducted in July 2010.^12^ The report is relevant in view of current environmental exposures witnessed due to ongoing illegal artisanal mining activities in Nigeria and other African countries.

**Abbreviations**
PDI: Potential oral daily intake

## Methods

Three free-range indigenous pullet chickens (A, B and C) were presented during a field investigation in July 2010 in the northwestern Nigerian state of Zamfara, where illegal small-scale gold mining activities caused the death of >500 children. The state of the chickens was raised as a concern by villagers while completing a questionnaire about other livestock concerns. The three chickens were obtained from the villagers and transported alive in chicken crates from the village to the local veterinary clinic, two hours away, located in the state capital, Gusau, for further clinical examination.

The chickens were humanely euthanized, and prepared for postmortem examination following standard procedures.^13^ Protocols were approved and performed in accordance with relevant local guidelines and regulations as set by the Animal Care and Use Committee of the National Veterinary Research Institute, Vom, Nigeria. Tissue samples were aseptically collected, washed in phosphate buffered saline and stored in sterile sample bottles. The kidneys, heart, brain and proventriculus were fixed in 10% neutral buffered formalin, processed and stained using Ziehl-Neelsen stain.^14^

The brain, bone, liver, kidneys, intestine, lungs and breast muscles were digested using the method described by the Association of Official Analytical Chemists Official Methods.^15^ Briefly, 0.2 g of each tissue sample was weighed and placed in Kjeldahl flasks into which 5 ml of nitric acid, 1 ml of perchloric acid and 0.5 ml sulphuric acid was added. The flask was heated at 200°C on a Kjeldahl heater until the solution became colorless and allowed to cool. The solution was made up to mark with deionized water in a 100 ml volumetric flask. The concentration of Pb in the digested samples was determined in a flame atomic absorption spectrophotometer (Shimadzu AA-6800, Shimadzu Corp, Japan), according to the manufacturer's specifications. Calibration with external standards was performed prior to analysis with a recovery of 91–93% and detection limit of 0.5 μg/g.

Biopsies of the liver and spleen were cultured both aerobically and anaerobically on blood and McConkey agar following standard procedures. The washed liver, spleen and intestine samples were prepared to constitute 80% suspension in phosphate buffered saline, centrifuged at 3000 rpm for 30 minutes and the supernatant collected as tissue extracts. Virus culture was carried out on the tissue extracts to check for hemagglutinating activity as described by Okwor *et al*.^16^

The potential oral daily intake (PDI) of lead by humans consuming tissues from the chickens was calculated as:

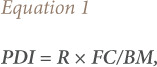
where, R is the concentration of lead in tissue, and FC is the rate of ingestion of food (g/day) consumed by an adult human with an average body mass (BM) of 60 kg. The FC was based on standard masses of food consumed per day by economically advantaged people on balanced diet. These were 100, 50 and 300 g/day for liver, kidneys and meat, respectively.^17^

## Results

**Figure 1:**
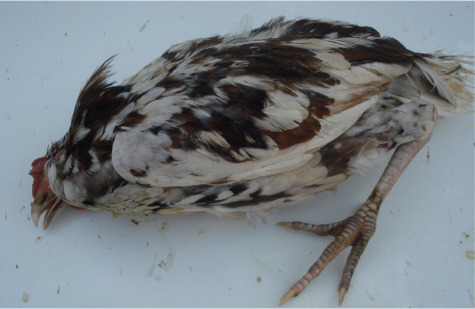
Chicken C, showing paresis of the limbs and unable to stand

The chickens were observed to be emaciated, weak, uncoordinated, showing staggering gait, torticollis, occasional cycling and were recumbent. The chickens showed paresis of the limbs and were unable to stand *(Figure 1).* On further enquiries, the residents claimed that several chickens, ducks and geese showed similar signs, and were either culled for meat or incinerated following death.

**Figure 2:**
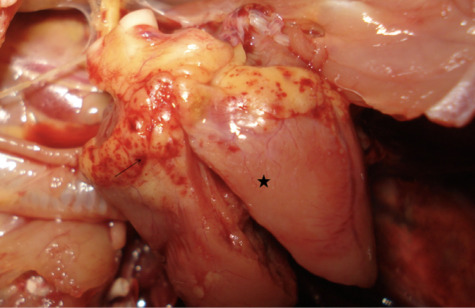
Chicken A. Heart showing petechial and ecchymotic hemorrhages on the coronary fat (arrow) and the myocardium with myocardial necrosis (star).

Grossly, chickens A, B and C had petechial and ecchymotic hemorrhages on the coronary fat and myocardium. The myocardium had diffuse areas of sharply demarcated pallor consistent with myocardial necrosis *(Figure 2).* The spleen was enlarged and congested. There was hepatic lipidosis, with gall bladder distention. The proventriculus had multifocal intramural dark areas of 0.2 cm in diameter, as seen from the serosa. Chicken C did not reveal visible gross lesions in the proventriculus. Chicken B carcass had two engorged amblyomma ticks at the base of the anterior part of the comb.

**Figure 3:**
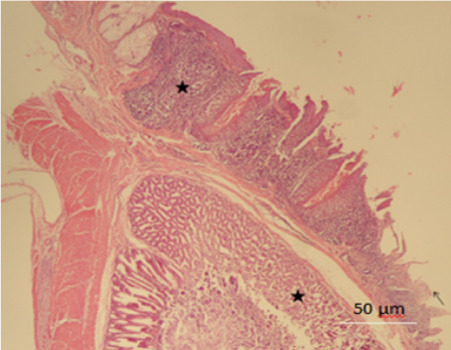
Chicken A. Proventriculus mucosal epithelium showing erosion and ulceration (arrow), with glandular necrosis (star) (hematoxylin-eosin stain; original magnification ×40; scale bar = 50 μm)

**Figure 4:**
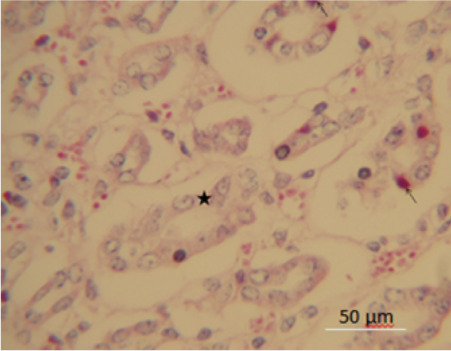
Chicken B. Renal tubular epithelia showing karyorrhexis, nuclei vacuolation (star) and hypereosinophilic cytoplasm (necrosis) with round eosinophilic intranuclear lead inclusions (arrows) (Ziehl-Neelsen stain; original magnification ×40; scale bar = 50 μm)

Histopathologically, for all the chickens, the gray matter of the cerebrum showed severe cortical lamina necrosis, the arterioles and venules were distorted and showed increased perivascular space. There was vasculitis and fibrinoid necrosis of myocardial vessels with extension to the myocardium. Proventricular mucosal and glandular epithelium was eroded, necrotic and ulcerated with focal fatty change *(Figure 3).* There was media hypertrophy and vasculitis of splenic arteriole within the periarteriolar lymphoid sheet. Renal tubular epithelia cells had karyorrhectic vacuolated nuclei and hypereosinophilic cytoplasm (necrosis). The tubular epithelial cells contained round eosinophilic intranuclear inclusions *(Figure 4)* with Ziehl-Neelsen stain, consistent with lead (Pb) poisoning.

Lead concentration in the tissues ranged between 7.5 mg/kg and 120.5 mg/kg wet weight *(Table 1).* The highest concentration of Pb was found in the thigh bones (120.5 mg/kg wet weight), while the intestines had the lowest (7.50 mg/kg wet weight). All tissue extracts were negative for Newcastle virus as there were no hemagglutinating activities in the allantoic cavities of inoculated embryos. Bacterial culture yielded Escherichia coli in the spleen and liver of all of the chickens. The PDI of Pb when consuming meat, liver and kidney from chicken is presented in Table 2. The estimated PDI was between 34.06–200.15 μg/day/kg body weight.

**Table 1:** Tissue Concentrations of Lead in Chickens Exposed to Artisanal Gold-Mining Activities in Zamfara, Nigeria

	**Chicken (mg/kg wet weight)**			
		
**Tissue type**	**A**	**B**	**C**	**Mean value**
Brain	87.00	75.05	100.10	87.38
Thigh bone	120.50	115.10	100.10	87.38
Liver	80.05	77.55	60.05	72.55
Kidneys	32.50	45.05	45.05	40.87
Intestines	23.70	35.05	7.50	22.08
Lungs	44.25	7.50	77.55	43.10
Breast muscle	72.55	35.05	12.50	40.03

**Table 2:** Potential Oral Daily Intake of Lead from Tissues of Lead-Poisoned Domestic Chickens in a Nigerian Community

**Tissue**	**PDI (μg Pb/kg body weight/day)^a^**
Liver	120.92
Kidney	34.06
Breast muscle	200.15

^a^Reference value of 3.57 μg/kg/day body mass.^18^

## Discussion

Lead poisoning in animals and humans is a major concern worldwide, due to the use of lead in many anthropogenic activities. In veterinary medicine, Pb poisoning is most common in canines, cattle and birds, but limited in other species due to reduced accessibility, selective eating habits or lower susceptibility. The main source of contamination for birds is oral ingestion of Pb from shotgun pellets.^6^ However, the natural environment may also be contaminated by Pb from anthropogenic sources such as mining.^6^ Lead contamination in the chickens was likely a result of the artisanal extraction of unusually lead-rich gold ore in the community. This type of gold mining uses rudimentary techniques of processing the gold ore in the residents' homes. Processing involves crushing with hammers or mortar and pestle, which are also used to grind the villagers' food. The women and children pound the ore into fragments for grinding in flour mills to separate the gold from the ore. As the rocks are pulverized, lead dust is spewed across the ground where the children play and chickens scavenge. After milling the ore, it is mixed with water and washed down a carpeted incline in a classic method called ‘sluicing’. This sluicing allows the heavy gold to stick to the fiber of the carpeting, thus forming a gravity concentrate.

The most common signs of lead intoxication across species are its effects on the nervous system. The impact on the central nervous system results in altered mentation, changes in responsiveness to environmental stimuli, loss of balance and the inability to stand.^19^ These signs were observed in the chickens examined in the current study. Anatomical malformations like unusual positions of the head and/neck were also observed in these chickens.

The present study found that Pb concentrations in the brain, bone, liver, kidneys, intestine and lungs of the chickens exceeded maximum residue limits in meat.^20^ The tissue Pb concentration was 75–1151 times more than the recommended limit of 0.5 mg/kg set by the European Commission.^20^ The high Pb concentration found in the bone compared to other examined tissues is suggestive of a case of chronic Pb poisoning, and may be due to the continuous displacement of calcium ions (Ca^2+^) from its binding sites. Gulson *et al*. reported that during acute Pb poisoning, concentrations of Pb will be higher in soft tissues, with levels higher in the kidneys than the liver.^21^ However, in chronic poisoning, the highest concentrations will be found in bone.

The histopathological lesions such as round intranuclear eosinophilic inclusions in the proximal tubular cells observed in this study are consistent with Pb toxicity in several studies. The significant concentrations of Pb and associated pathology in the cerebrum may be associated with the nervous system signs observed in these birds. It appears that the substantial Pb levels found in the soil, water and vegetation within the community contributed considerably to the high values recorded in the tissues of the chickens.^12^,^22^,^23^

The E. coli isolated in the spleen and liver of all the chickens are considered to be opportunistic contaminants and may have contributed to the degree of pathology observed in these organs, since Pb is known to be immunosuppressive.^24^ A study suggested that the presence of Pb may alter immune competence against E. coli and other bacteria.^25^ Escherichia coli is reported to have a high prevalence rate (up to 81%) and is a common opportunistic enterobacteria in chickens in Nigeria.^26^ Clinical signs of colibacillosis are respiratory distress, sneezing, coughing, conjunctivitis, nasal and ocular discharge, poor growth and death.^27^ Gross lesions in colibacillosis include, but are not limited to, catarrhal exudates in trachea and bronchi, congestion of trachea mucosal membrane, splenomegaly, hepatomegaly and air sacculitis.^27^ The findings of the current report indicates that although E. coli was isolated in the chickens' spleen and liver, the signs presented do not indicate E. coli infection. The infection could aggravate the symptoms of Pb, but its exact role in the symptoms observed could not be deduced in this report.

The virology result which was negative for Newcastle virus antigens ruled out the possibility of Newcastle disease, which can also produce the neurological signs observed in this study. Another viral disease which presents with similar symptoms is avian encephalomyelitis. The disease affects chicks with clinical signs of ataxia, tremor, weakness, and epidemic tremors which progress to paralysis and recumbency.^28^ However, avian encephalomyelitis was ruled out considering the age of the chickens and the absence of epidemic tremors pathognomonic to the disease.

Although we were unable to determine how much poultry products such as eggs and meat contribute to the total dietary intake of an individual in the Zamfara community, estimates of PDI values are approximately 10-57 times greater than what is considered normal intake levels of 3.57 μg/kg/day body mass.^18^ The PDI estimate did not take into account Pb residues in the entire environment. It can be inferred that the consumption of chicken from the contaminated communities poses a significant health risk and is a public health concern. Despite the high levels of Pb in the chickens, they are still a small contributor to the total daily intake of Pb from all other sources for children and adults in the community.

## Conclusions

It is highly likely that the chickens were intoxicated with Pb, based on prior history, tissue Pb concentrations and pathology. This calls for concerted efforts in eliminating sources of environmental pollution from the gold mining activity in these communities, due to the public health risks posed by Pb accumulation in tissues of animals which then moves into the food chain. We recommend long-term continuous biomonitoring of domestic animals in these communities to determine if bioremediation measures are successful, in view of the paucity of data on Pb burden in animals in these communities. Poisoning in animal populations may serve as a sentinel to assess the extent of environmental contamination and human health problems related to Pb. Findings from further studies would ensure remediation through proper government policies. The role of education and partnership with government and non-governmental organizations cannot be overemphasized.
